# Knowledge of State Gun Laws Among US Adults in Gun-Owning Households

**DOI:** 10.1001/jamanetworkopen.2021.35141

**Published:** 2021-11-18

**Authors:** Ali Rowhani-Rahbar, Miriam J. Haviland, Deborah Azrael, Matthew Miller

**Affiliations:** 1 Department of Epidemiology, School of Public Health, University of Washington, Seattle; 2 Firearm Injury and Policy Research Program, University of Washington, Seattle; 3 Harvard Injury Control Research Center, Harvard T.H. Chan School of Public Health, Harvard University, Boston, Massachusetts; 4 Department of Health Sciences, Bouvé College of Health Sciences, Northeastern University, Boston, Massachusetts

## Abstract

This survey study assesses US gun owners’ knowledge of their state’s gun laws.

## Introduction

Gun laws are intended to reduce firearm-related harm. Research on the effectiveness of these laws has yielded mixed findings. Supporting evidence on the effectiveness of a few gun laws exists; however, for most gun laws, the evidence is either inconclusive or lacking.^1^ A lack of awareness of state gun laws or the erroneous belief that these laws are not in force could mean that the laws do not have their intended impact. This scenario is especially pertinent when the knowledge of gun laws is likely integral to behavioral change. Previous research has assessed public support for gun laws^2^ but not people’s knowledge of specific gun laws that are in force in their state of residence.

## Methods

In this survey study, we analyzed data from the 2019 National Firearms Survey (July 30 to August 11, 2019), which comprised adults living in gun-owning households. Respondents were sampled from a probability-based web panel of 55 000 noninstitutionalized, English-speaking adults who were representative of the US population.^3^ Respondents self-reported race and ethnicity (Hispanic, non-Hispanic Black, non-Hispanic White, non-Hispanic 2 or more races, or non-Hispanic other [American Indian or Alaska Native, Asian, and Native Hawaiian or Pacific Islander]) in Ipsos’ annual KnowledgePanel. The analysis included a random sample of 6721 individuals who lived in a gun-owning household from 50 states and the District of Columbia. Of these individuals, 4379 completed the survey (a completion rate of 65.2%), and 4030 (60.0%) were qualified completers (those 18 years or older and not currently active duty in the US Armed Forces). Completion of the online survey was considered to be informed consent for study participation. The Harvard T.H. Chan School of Public Health’s Institutional Review Board approved the study. We followed the American Association for Public Opinion Research (AAPOR) reporting guideline.

Respondents were asked whether they personally owned a gun and, to the best of their knowledge, whether particular gun laws were in place in their state (the Supplement). We reviewed penal codes to ascertain whether state gun laws were operational at the time of the survey.^4,5^ Poststratification weights were adjusted for nonresponse and undercoverage or overcoverage from the study-specific sample design compared with expected demographic distributions from the US Census Bureau’s Current Population Survey and American Community Survey. Weights for gun ownership came from KnowledgePanel data supplied by the research firm Ipsos. Analyses were conducted using SAS, version 9.4 (SAS Institute).

## Results

Of the 4030 qualified respondents (mean [SD] age, 50.3 [17.0] years), 52% were men, and 77% were non-Hispanic White (Table 1). The proportion of gun owners who correctly believed that their state had a specific gun law ranged from 36.7% (95% CI, 33.4%-39.9%) for child access prevention laws to 64.0% (95% CI, 60.2%-67.9%) for laws that required reporting lost or stolen guns (Table 2). Among gun owners, 15.3% (95% CI, 12.7%-17.9%) incorrectly believed that their state did not have a child access prevention law, and 11.0% (95% CI, 8.6%-13.4%) incorrectly believed that their state did not require a background check for private firearm sales. In addition, 55.5% (95% CI, 52.0%-59.1%) of gun owners and 75.0% (95% CI, 70.2%-79.7%) of non–gun owners were unsure if their state had an extreme risk protection order law. Anywhere from at least 29.0% (95% CI, 25.5%-32.5%) to 74.5% (95% CI, 70.4%-78.6%) of respondents were unsure if their state had a child access prevention law, a law that required background checks before private firearm sales, or a law that required reporting lost or stolen guns to the police.

**Table 1.** Characteristics of 2019 National Firearms Survey Respondents

Characteristic	Weighted % (95% CI)^a^		
	Total (n = 4030)	Gun owners (n = 2950)	Non–gun owners (n = 1080)
Age, y			
18-29	16.0 (14.2-17.8)	12.4 (10.7-14.3)	23.5 (19.8-27.1)
30-44	22.8 (21.2-24.4)	23.1 (21.2-25.1)	22.2 (19.3-25.0)
45-59	28.1 (26.4-29.7)	29.9 (28.0-31.9)	24.2 (21.3-27.1)
≥60	33.1 (31.5-34.7)	34.5 (32.6-36.4)	30.2 (27.2-33.2)
Sex			
Male	52.0 (50.2-53.9)	69.5 (67.5-71.5)	15.7 (12.6-18.8)
Female	48.0 (46.1-49.8)	30.5 (28.5-32.5)	84.3 (81.2-87.4)
Race and ethnicity^b^			
Hispanic	10.2 (8.8-11.6)	9.4 (7.8-10.9)	11.9 (9.1-14.7)
Non-Hispanic Black	7.6 (6.5-8.7)	8.0 (6.7-9.4)	6.8 (4.9-8.7)
Non-Hispanic White	76.9 (75.1-78.7)	77.5 (75.4-79.6)	75.6 (72.2-79.0)
Non-Hispanic 2 or more races	1.8 (1.3-2.3)	1.7 (1.2-2.1)	2.1 (1.1-3.1)
Non-Hispanic other^c^	3.5 (2.6-4.4)	3.4 (2.3-4.5)	3.6 (2.0-5.3)
Marital status			
Married or partnered	74.7 (73.0-76.5)	74.5 (72.5-76.4)	75.3 (71.8-78.8)
Divorced, separated, or widowed	11.5 (10.4-12.5)	14.1 (12.8-15.5)	5.9 (4.4-7.5)
Never married	13.8 (12.2-15.4)	11.4 (9.7-13.0)	18.8 (15.4-22.2)
Educational level			
Bachelor’s degree or higher	30.9 (29.3-32.4)	31.8 (29.9-33.7)	28.8 (25.9-31.7)
<Bachelor’s degree	69.1 (67.6-70.7)	68.2 (66.3-70.1)	71.2 (68.3-74.10
Households with at least 1 child	32.6 (30.8-34.5)	31.3 (29.2-33.5)	35.4 (31.8-38.9)
Age of children, y			
0-5	40.6 (37.1-44.1)	40.1 (35.9-44.3)	41.5 (35.1-47.9)
6-12	48.2 (44.6-51.9)	51.1 (46.8-55.4)	43.0 (36.5-49.5)
13-17	44.5 (40.8-48.1)	41.8 (37.5-46.1)	49.4 (42.8-56.0)
Residence in metropolitan statistical area			
Yes	79.5 (78.0-81.1)	79.5 (77.7-81.3)	79.5 (76.5-82.4)
No	20.5 (18.9-22.0)	20.5 (18.7-22.3)	20.5 (17.6-23.5)
US region			
Northeast	11.9 (10.7-13.0)	11.2 (9.9-12.5)	13.3 (11.0-15.7)
Midwest	23.6 (22.0-25.1)	22.4 (20.6-24.2)	26.0 (22.9-29.0)
South	42.3 (40.4-44.1)	43.7 (41.5-45.9)	39.2 (35.7-42.7)
West	22.3 (20.7-23.9)	22.7 (20.8-24.5)	21.5 (18.6-24.4)
Veteran status			
Yes	13.8 (12.7-15.0)	19.2 (17.6-20.8)	2.6 (1.6-3.8)
No	86.2 (85.0-87.3)	80.8 (79.2-82.4)	97.3 (96.2-98.4)

^a^ Percentages in some sections do not add to 100% because of rounding.

^b^ Race and ethnicity were self-reported by respondents to Ipsos’s KnowledgePanel based on predefined categories: American Indian or Alaska Native; Asian; Black or African American; Hispanic, including Cuban or Cuban American, Mexican, Mexican American, Chicano, Puerto Rican, or other Spanish or Hispanic or Latino; Native Hawaiian or Pacific Islander; White; or 2 or more races. Ipsos then provided the data for this study as listed in the categories in the table.

^c^ Other included the following races and ethnicities: American Indian or Alaska Native, Asian, and Native Hawaiian or Pacific Islander.

**Table 2.** 2019 National Firearms Survey Respondents’ Knowledge of Gun Laws in Their State of Residence

State law	Weighted % (95% CI)^a^					
	Gun owners (n = 2950)			Non–gun owners (n = 1080)		
	Yes, state has law	No, state does not have law	Unsure if state has law	Yes, state has law	No, state does not have law	Unsure if state has law
Background check before sale of a gun transferred by someone other than a licensed dealer						
No, state does not have law	45.0 (42.3-47.7)	23.8 (21.5-26.0)	31.3 (28.9-33.6)	40.0 (35.8-44.3)	12.1 (9.3-14.9)	47.8 (43.5-52.2)
Yes, state does have law	60.0 (56.2-63.8)	11.0 (8.6-13.4)	29.0 (25.5-32.5)	55.4 (49.1-61.6)	4.7 (2.6-6.7)	39.9 (33.7-46.2)
Reporting lost or stolen guns to the police by firearm owner						
No, state does not have law	44.7 (42.1-47.3)	6.2 (5.0-7.4)	49.1 (46.5-51.7)	32.8 (28.8-36.8)	1.8 (0.7-2.9)	65.4 (61.4-69.4)
Yes, state does have law	64.0 (60.2-67.9)	2.2 (1.1-3.3)	33.8 (30.0-37.5)	42.5 (36.0-48.9)	0.9 (0.1-1.7)	56.6 (50.2-63.1)
Child access prevention						
No, state does not have law	18.1 (15.8-20.4)	22.4 (19.9-24.8)	59.5 (56.6-62.4)	10.0 (7.3-12.6)	15.5 (12.0-19.0)	74.5 (70.4-78.6)
Yes, state does have law	36.7 (33.4-39.9)	15.3 (12.7-17.9)	48.1 (44.7-51.4)	21.5 (17.3-25.8)	8.3 (5.7-10.9)	70.1 (65.4-74.9)
Extreme risk protection order						
No, state does not have law	22.3 (19.9-24.6)	11.0 (9.2-12.8)	66.7 (64.1-69.4)	10.4 (7.7-13.1)	5.1 (3.3-6.9)	84.5 (81.4-87.6)
Yes, state does have law	40.1 (36.6-43.6)	4.4 (2.9-5.8)	55.5 (52.0-59.1)	22.5 (18.0-26.9)	2.6 (0.6-4.6)	75.0 (70.2-79.7)

^a^ Percentages in some sections do not add to 100% because of rounding.

## Discussion

Despite wide acceptance that interrogating causal pathways should play a critical role in policy evaluations,^6^ few published studies in the gun literature have taken this approach. That many US adults in gun-owning households have limited knowledge of the gun laws in their state of residence should be considered in interpreting the evaluations of potential harm-reducing benefits of firearm legislation. This consideration is especially pertinent for evaluations of laws similar to those in this investigation that plausibly affect behavior when individuals in gun-owning households are aware that the laws are in force. The findings of the present study suggest the need to promote further awareness about state gun laws, especially among individuals who live in gun-owning households. This study is limited by potential reporting bias.

## Notes

Supplement. **eAppendix.** Survey Questions

## References

[1] RAND Corporation. Gun policy in America. Updated April 2021. Accessed June 22, 2021. https://www.rand.org/research/gun-policy.html

[2] Barry CL, Webster DW, Stone E, Crifasi CK, Vernick JS, McGinty EE. Public support for gun violence prevention policies among gun owners and non-gun owners in 2017. Am J Public Health. 2018;108(7):878-881. doi:10.2105/AJPH.2018.304432 29771617 PMC5993389

[3] Salhi C, Azrael D, Miller M. Patterns of gun owner beliefs about firearm risk in relation to firearm storage: a latent class analysis using the 2019 National Firearms Survey. Inj Prev. 2020;27:271-276. doi:10.1136/injuryprev-2019-043624 32665253

[4] Giffords Law Center. Gun laws. Accessed June 28, 2021. https://giffords.org/lawcenter/gun-laws

[5] Cherney S, Morral AR, Schell TL, Smucker S. Development of the RAND state firearm law database and supporting materials. RAND Corporation. Updated September 4, 2020. Accessed June 22, 2021. https://www.rand.org/pubs/tools/TLA243-2.html

[6] Ludwig J, Kling JR, Mullainathan S. Mechanism experiments and policy evaluations. J Econ Perspect. 2011;25(3):17-38. doi:10.1257/jep.25.3.17

