# Supplementary material for: Knowledge of State Gun Laws Among US Adults in Gun-Owning Households
Source: JAMA Netw Open. 2021 Nov 18;4(11):e2135141. doi: 10.1001/jamanetworkopen.2021.35141 (PMC8603069; doi:10.1001/jamanetworkopen.2021.35141)
Supplement: Supplement. — eAppendix. Survey Questions [file jamanetwopen-e2135141-s001.pdf]

Supplementary Online Content

Rowhani-Rahbar A, Haviland MJ, Azrael D, Miller M. Knowledge of state gun laws among US adults in gun-owning households. *JAMA Netw Open*. 2021;4(11):e2135141. doi:10.1001/jamanetworkopen.2021.35141

eAppendix. Survey Questions

This supplementary material has been provided by the authors to give readers additional information about their work.

© 2021 Rowhani-Rahbar A, et al. *JAMA Netw Open*.

Survey Questions

The next questions are about working guns. Throughout this survey we use the word "gun" to refer to any firearm, including pistols, revolvers, shotguns, and rifles, but not including air guns, bb guns, starter pistols or paintball guns. By working guns, we mean guns that are in working order and capable of being fired.

Do you or does anyone else you live with currently own any type of gun?
1. Yes
2. No

Do you personally own a gun?
1. Yes
2. No

Other than yourself, does anyone else in your household own a gun?
1. Yes
2. No
3. Don't know

Many states have laws about buying, selling, carrying, and storing firearms. The next questions ask about whether, to the best of your knowledge, particular laws are in place in the state you currently live in.

To the best of your knowledge, does the state you currently live in have a law that imposes penalties on gun owners whose guns could be or are accessed by children? These laws, sometimes called Child Access Prevention laws or Safe Storage laws, may or may not require that a child's access results harm to self or others.
1. Yes
2. No
3. Don't know

Does the state you currently live in have a law requiring a background check prior to the sale of a gun that is transferred by someone other than a licensed dealer?
1. Yes
2. No
3. Don't know

Does the state you currently live in require firearms owners to report lost or stolen guns to the police?
1. Yes
2. No
3. Don't know

Does the state you currently live in have a law that allows law enforcement, family members and/or others to ask a judge to temporarily remove guns if a person is a danger to themselves or others and are at a high risk of committing violence with a firearm (sometimes called ERPO, GVRO or Red Flag laws)?
1. Yes
2. No
3. Don't know

© 2021 Rowhani-Rahbar A, et al. *JAMA Netw Open*.
